# Endothelial nitric oxide synthase gene polymorphisms and risk of diabetic nephropathy: a systematic review and meta-analysis

**DOI:** 10.1186/1471-2350-15-9

**Published:** 2014-01-16

**Authors:** Bruno Schmidt Dellamea, Lana Catani Ferreira Pinto, Cristiane Bauermann Leitão, Katia Gonçalves Santos, Luis Henrique Santos Canani

**Affiliations:** 1 Endocrine Division, Hospital de Clínicas de Porto Alegre, Ramiro Barcelos 2350, CEP 90035-903 Porto Alegre, RS, Brazil; 2 Universidade Luterana do Brasil, Canoas, Brazil

## Abstract

**Background:**

Nitric oxide (NO) has numerous functions in the kidney, including control of renal and glomerular hemodynamics, by interfering at multiple pathological and physiologically critical steps of nephron function. Endothelial NOS (eNOS) gene has been considered a potential candidate gene to diabetic nephropathy (DN) susceptibility. Endothelial nitric oxide synthase gene (eNOS-3) polymorphisms have been associated with DN, however some studies do not confirm this association. The analyzed polymorphisms were 4b/4a, T-786C, and G986T.

**Methods:**

The Preferred Reporting Items for Systematic Reviews and Meta-Analysis (PRISMA) statement was used in this report. Case–control studies that had diabetic patients with DN as cases and diabetic patients without nephropathy as controls, as well as that evaluated at least one of the three polymorphisms of interest were considered eligible. All studies published up until December 31^st^, 2012 were identified by searching electronic databases. Hardy-Weinberg equilibrium assessment was performed. Gene-disease association was measured using odds ratio estimation based on the following genetic contrast/models: (1) allele contrast; (2) additive model; (3) recessive model; (4) dominant model and (4) co-dominant model.

**Results:**

Twenty-two studies were eligible for meta-analysis (4b/a: 15 studies, T-786C: 5 studies, and G984T: 12 studies). Considering 4b/a polymorphism, an association with DN was observed for all genetic models: allele contrast (OR = 1.14, CI: 1.04-1.25); additive (OR = 1.77, CI: 1.37-2.28); recessive (OR = 1.77, CI: 1.38-2,27); dominant (OR = 1.12, CI: 1.01-1.24), with the exception for co-dominance model. As well, T-786C polymorphism showed association with all models, with exception for co-dominance model: allele contrast (OR = 1.22, CI: 1.07-1.39), additive (OR = 1.52, CI: 1.18-1.97), recessive (OR = 1.50, CI: 1.16-1.93), and dominant (OR = 1.11, CI: 1.01-1.23). For the G894T polymorphism, an association with DN was observed in allelic contrast (OR = 1.12, CI: 1.03-1.25) and co-dominance models (OR = 1.13, CI: 1.04-1.37).

**Conclusions:**

In the present study, there was association of DN with eNOS 4b/a and T-786C polymorphism, which held in all genetic models tested, except for co-dominance model. G894T polymorphism was associated with DN only in allele contrast and in co-dominance model. This data suggested that the eNOS gene could play a role in the development of DN.

## Background

Nitric oxide (NO) is a short-lived gaseous lipophilic molecule produced in almost all tissues and organs [1,2]. It is a free radical that exerts a variety of biological actions under both physiological and pathological conditions [3]. NO is formed from its precursor L-arginine by a family of NO synthases (NOSs). NOS system consists of three distinct isoforms, encoded by three distinct genes, including neuronal (nNOS or NOS-1), inducible (iNOS or NOS-2), and endothelial (eNOS or NOS-3). The gene encoding eNOS is located on chromosome 7 (7q35-q36) and contains 26 exons, with an entire length of 21 kb [3,4].

NO has numerous functions in the kidney, including control of renal and glomerular hemodynamics, by interfering at multiple pathological and physiologically critical steps of nephron function. NO dilates both the afferent and the efferent arteriole, augmenting the glomerular filtration rate (GFR) and influencing renal sodium handling [5]. NO also mediates pressure natriuresis, maintenance of medullary perfusion, decrease of tubuloglomerular reabsorption, and modulation of renal sympathetic nerve activity [6]. The net effect of NO in the kidney is to promote natriuresis and diuresis, along with renal adaptation to dietary salt intake [7,8].

eNOS gene has been considered a potential candidate gene to diabetic nephropathy (DN) susceptibility. Since 1998, several polymorphisms of the eNOS gene have been identified, and their association with various diseases has been explored. Three polymorphisms have been the subject of research in relation to DN, however the results are highly variable. The polymorphisms potentially associated with DN are a 27-bp repeat in intron 4 (VNTR), the T-786C single nucleotide polymorphism (SNP) in the promoter region (rs2070744), and G894T missense mutation in exon 7 (rs1799983) [9]. Some of these polymorphisms are associated with reduction of either eNOS activity (-786C in the promoter area) or plasma concentrations of NO (four repeats in intron 4) [2].

However, the potential association of eNOS gene variants with the induction and progression of DN remains controversial. Some authors found a higher frequency of eNOS polymorphisms in patients with end-stage renal disease (ESRD) and DN [10-17], but not all studies reported this association [18-20].

The objective of the present study was to evaluate if eNOS gene polymorphisms are associated with DN through a systematic review of the literature and a meta-analysis.

## Results and discussion

**Figure 1:**
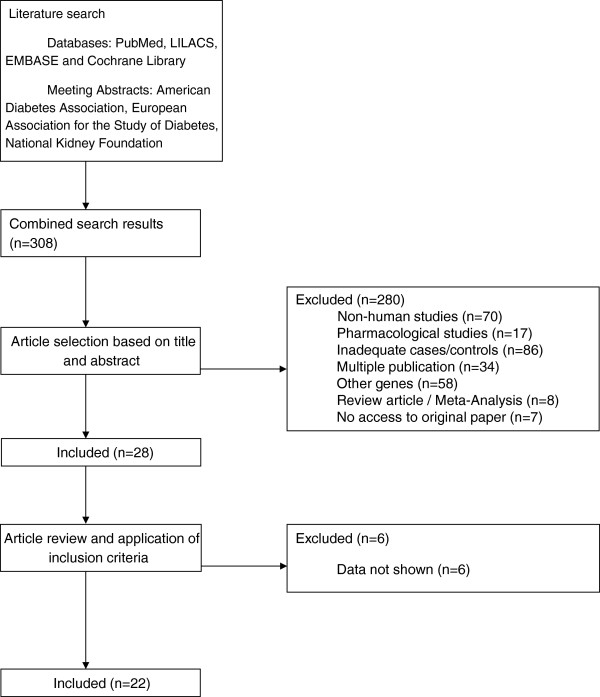
Search strategy.

Three-hundred and nine studies were identified, and 281 were excluded based on review of titles and abstracts (70 animal experimental studies, 17 pharmacological studies, 86 without adequate cases or controls, 58 without the genes or polymorphisms of interest, 3 review articles, 5 meta-analysis, 35 studies with multiple publications of the same data presented with different titles, 7 no accesses to original data even after contacting authors). Twenty-eight articles were eligible and had the full text evaluated. Six studies were excluded due to lack of information regarding genotypic distribution. A total of 22 studies fulfilled the eligible criteria and were included for the meta-analysis (Figure 1).

Clinical characteristics of individual studies are described in Table 1. Regarding quality assessment, the phenotype definitions as cases or controls were appropriated, but none of the studies included information if genotyping was performed by personnel blinded to clinical status. Of the 22 studies included, 15 provided 4054/3405 cases/controls for 4b/a; 5 provided 1436/1286 cases/controls for T-786C; and 12 provided 3316/2765 cases/controls for G894T. The allelic frequency of 4b, T-786, and G894 in cases/controls was 6647/5702, 1863/1795, and 4691/4017 respectively (Table 2).

**Table 1:** Baseline studies characteristics

**Author**	**Year**	**Polymorphism**	**Type of DM**	**Ethnicity**	**Cases/controls (n)**	**Criteria**	**Male/female (%)**	**Age**	**DM duration (years)**
Ahluwalia et al. [21]	2008	G894T, 4a/b, T-786C	2	East Asians	Case (195)	Overt proteinuria	35/65	60.0 ± 6.15	16.5 ± 6.3
					Control (255)	Normoalbuminuria	41/59	60.5 ± 5.7	15.6 ± 5.2
Bessa et al. [22]	2011	G894T	2	African	Case (40)	Albuminuria > 30 mg/24 h	21/19	58.8 ± 12.5	19.4 ± 4.2
					Control (40)	Albuminuria < 30 mg/24 h	17/23	55.4 ± 8.8	15.3 ± 3.7
Cai et al. [23]	1998	G894T	2	Whites	Case (116)	Microalbuminuria	NA	NA	NA
					Control (284)	Normoalbuminuria	NA	NA	NA
Degen et al. [24]	2001	4a/b	1and 2	Whites	Case (207)	AER >30 mg/24 h	NA	NA	>10 yrs
					Control (418)	AER <30 mg/24 h	NA	NA	>10 yrs
Ezzidi et al. [10]	2008	G894T, 4a/b, T-786C	2	African	Case (515)	AER >30 mg/24 h	46/54	59.6 ± 10.8	13.5 ± 6.3
					Control (402)	AER <30 mg/24 h	42/58	59.1 ± 11.2	11.5 ± 6.2
Fujita et al. [25]	2000	4a/b	2	East Asians	Case (102)	AER >200 mcg/min	60/40	61.0 ± 21.0	NA
					Control (65)	AER <20 mcg/min	46/54	62.0 ± 10.0	NA
Ksiasek et al. [13]	2003	4a/b	2	Whites	Case (178)	With DN	48/52	57.9 ± 8.2	8.7 ± 3.1
					Control (232)	Without DN	51/49	58.3 ± 6.8	8.0 ± 2.6
Lin et al. [25]	2002	4a/b	2	East Asians	Case (80)	With DN	NA	NA	NA
					Control (48)	Normoalbuminuria	NA	NA	NA
Mollsten et al. [26]	2006	G894T, 4a/b	1	Whites	Case (955)	AER >20 mcg/min	58/42	40.3 ± 10.0	28 (5–65)
					Control (555)	AER <20 mcg/min + DM duration >20 yrs	41/59	42.2 ± 10.2	28 (20–57)
Mollsten et al. [18]	2009	G894T	1	Whites	Case (458)	AER >300 mg/24 h	39/61	42.0 ± 10.4	27 (7–65)
					Control (319)	AER <30 mg/24 h	55/45	43.7 ± 11.0	23 (15–63)
Neuguebauer et al. [14]	2000	4a/b	2	East Asians	Case 1 (104)	AER 20–200 mg/g Cr	53/47	59.0 ± 11.1	13.8 ± 5.1
					Case 2 (39)	AER >200 mg/g Cr	74/26	59.0 ± 8.6	15.2 ± 4.5
					Control (82)	AER <20 mg/g Cr	65/35	56.0 ± 8.6	13.3 ± 4.5
Rahimi et al. [27]	2012	G894T	2	West Asians	Case 1 (68)	Albumin to creatinin ratio >300 mg/g	33/35	57.1 ± 8.7	11.1 ± 6.4
					Case 2 (72)	Albumin to creatinin ratio 30–299 mg/g	23/46	55.3 ± 8.6	8.6 ± 5.2
					Control (72)	Albumin to creatinin ratio <30 mg/g	23/49	54.4 ± 7.9	7.7 ± 5.4
Rippin et al. [28]	2003	4a/b	1	Whites	Case (464)	Overt proteinuria	NA	NA	NA
					Control (396)	Normoalbuminuria	NA	NA	NA
Santos et al. [29]	2009	G894T, 4a/b, T-786C	2	Whites	Case (376)	AER >20 mcg/min or >17 mg/dl	57/43	60.4 ± 9.7	15.0 ± 9.1
					Control (268)	AER <20 mcg/min or <17 mg/dl	37/63	62.0 ± 9.4	16.7 ± 6.8
Shestakova et al. [16]	2006	4a/b	1	Whites	Case (63)	AER >300 mg/24 h	47/53	25.7 ± 6.4	12.6 ± 2.8
					Control (66)	AER <30 mg/24 h	37/63	40.8 ± 10.2	26.8 ± 6.9
Shimizu et al. [30]	2002	4a/b	2	East Asian	Case 1 (107)	Overt proteinuria	70/30	63.1 ± 10.6	15.5 ± 11.0
					Case 2 (124)	Overt proteinuria + Cr >1.5 mg/dl	75/25	65.1 ± 8.8	19.8 ± 7.8
					Control (203)	Normoalbuminuria > DM >10 yrs	65/35	63.7 ± 8.8	18.6 ± 7.8
Shin Shin et al. [31]	2004	G894T	2	East Asians	Case 1 (35)	Microalbuminuria	46/54	62.9 ± 10.9	16 (12–20)
					Case 2 (83)	Overt proteinuria	46/54	58.8 ± 9.7	16 (11–20)
					Control (59)	Normoalbuminuric	25/75	61.6 ± 11.7	12 (10–16)
Shoukry et al. [32]	2012	G894T, 4a/b, T-786C	2	African	Case	Albumin to creatinin ratio >300 mg/g	108/92	55.3 ± 5.8	14.5 ± 4.3
					Control	Albumin to creatinin ratio <30 mg/g	116/84	54.6 ± 5.2	13.8 ± 3.2
Tamemoto et al. [33]	2008	G894T	NA	East Asians	Case (124)	Microalbuminuria	NA	NA	NA
					Control (211)	Normoalbuminuria	NA	NA	NA
Taniwaki et al. [20]	2001	4a/b	2	East Asians	Case 1 (44)	Microalbuminuria	59/41	60.5 ± 8.5	10.9 ± 7.4
					Case 2 (22)	Overt proteinuria	68/32	59.0 ± 10.5	12.8 ± 6.5
					Case 3 (20)	Overt proteinuria + Cr >1.5 mg/dl	50/50	64.2 ± 7.8	19.1 ± 9.7
					Control (69)	Normoalbuminuria	59/41	60.1 ± 9.8	7.4 ± 4.5
Tiwari et al. [19]	2009	G894T	2	South Asians	Case 1 (90)	DM >2 yrs + Cr >2 mg/dl from N India	87/13	53.6 ± 11.0	9.6 ± 6.8
					Case 2 (106)	DM >2 yrs + Cr >2 mg/dl from S India	76/24	55.9 ± 11.5	14.0 ± 6.4
					Control 1 (75)	DM >10 yrs + Cr <2 mg/dl from N India	53/47	61.0 ± 8.9	15.4 ± 8.1
					Control 2 (149)	DM >10 yrs + Cr <2 mg/dl from S India	68/32	60.5 ± 11.4	15.5 ± 6.91
Zanchi et al. [17]	2000	4a/b, T-786C	1	Whites	Case 1 (74)	AER >200 mcg/mg	42/58	35.5 ± 7.3	24.9 ± 9.0
					Case 2 (78)	AER >200 mcg/mg + Cr >1.5 mg/dl	49/51	35.7 ± 6.5	24.5 ± 6.8
					Control (195)	AER <20 mcg/mg + DM >15 yrs	52/48	36.5 ± 7.6	23.7 ± 6.3

Where: AER : albumin excretion rate; DM: diabetes mellitus; DN: diabetic nephropathy; NA: not available; Cr: creatinine.

**Table 2:** Polymorphisms distribution

**Author**	**Distribution of the T-786C polymorphism**						**HWE**
	**Cases**			**Controls**			**p value**
	**TT**	**TC**	**CC**	**TT**	**TC**	**CC**	
Ahluwalia et al. [21]	121	62	12	165	87	3	0.020
Ezzidi et al. [10]	261	215	34	224	139	32	0.115
Santos et al. 2011 [29]	140	160	76	93	104	44	0.138
Shoukry et al. 2012 [32]	57	89	54	84	83	33	0.129
Zanchi et al. [17]	57	65	30	75	100	20	0.123
	**Distribution of the G894T polymorphism**						**HWE**
	**Cases**			**Controls**			**p value**
	**GG**	**GT**	**TT**	**GG**	**GT**	**TT**	
Ahluwalia et al. [21]	82	81	32	125	105	25	0.658
Bessa et al. 2011 [22]	10	18	12	17	19	4	1.000
Cai et al. [23]	65	44	7	148	109	27	0.310
Ezzidi et al. [10]	185	247	81	165	195	41	0.151
Mollsten et al. [34]	492	365	89	268	232	51	0.919
Mollsten et al. [18]	293	133	32	182	121	16	0.540
Rahimi et al. 2012 [27]	68	45	13	39	17	7	**0.038**
Santos et al. 2011 [29]	176	166	32	118	95	22	0.640
Shin Shin et al. [31]	95	23	0	52	7	0	1.000
Shoukry et al. 2012 [32]	66	94	40	99	77	24	0.140
Tamemoto et al. [33]	104	18	2	181	27	3	0.117
Tiwari et al. [19]	82	21	3	91	43	13	**0.035**
	**Distribution of the 4b/4a polymorphism**						**HWE**
	**Cases**			**Controls**			**p value**
	**bb**	**ba**	**aa**	**bb**	**ba**	**aa**	
Ahluwalia et al. [21]	146	28	21	189	61	5	1.000
Degen et al. [24]	229	94	4	297	105	9	1.000
Ezzidi et al. [10]	314	162	29	234	143	21	1.000
Fujita et al. [25]	81	21	0	55	10	0	1.000
Ksiasek et al. 2003 [13]	105	58	15	147	66	19	**0.007**
Lin et al. [26]	115	21	1	41	6	1	0.271
Mollsten et al. [34]	656	248	39	389	145	19	0.220
Neugebauer et al. [14]	101	26	6	71	10	1	0.351
Rippin et al. [28]	344	108	12	297	90	9	0.519
Santos et al. 2011 [29]	237	99	11	168	59	5	1.000
Shestakova et al. [16]	14	48	1	34	31	1	0.052
Shimizu et al. [30]	180	44	6	156	44	3	1.000
Shoukry et al. 2012 [32]	124	64	12	131	60	9	0.502
Taniwaki et al. [20]	63	21	2	50	19	0	0.340
Zanchi et al. [17]	80	27	37	144	47	4	1.000

HWE (Hardy-Weinberg equilibrium).

Hardy-Weinberg equilibrium (HWE) was assessed using exact test and P-value < 0.05 were considered significant. Only 4 studies (1 study for T-786C; 2 for G984T; and 1 for 4b/a) with controls were not in HWE (Table 2). These studies were subjected to a sensitive analysis, and their exclusion did not show significant difference on OR.

**Figure 2:**
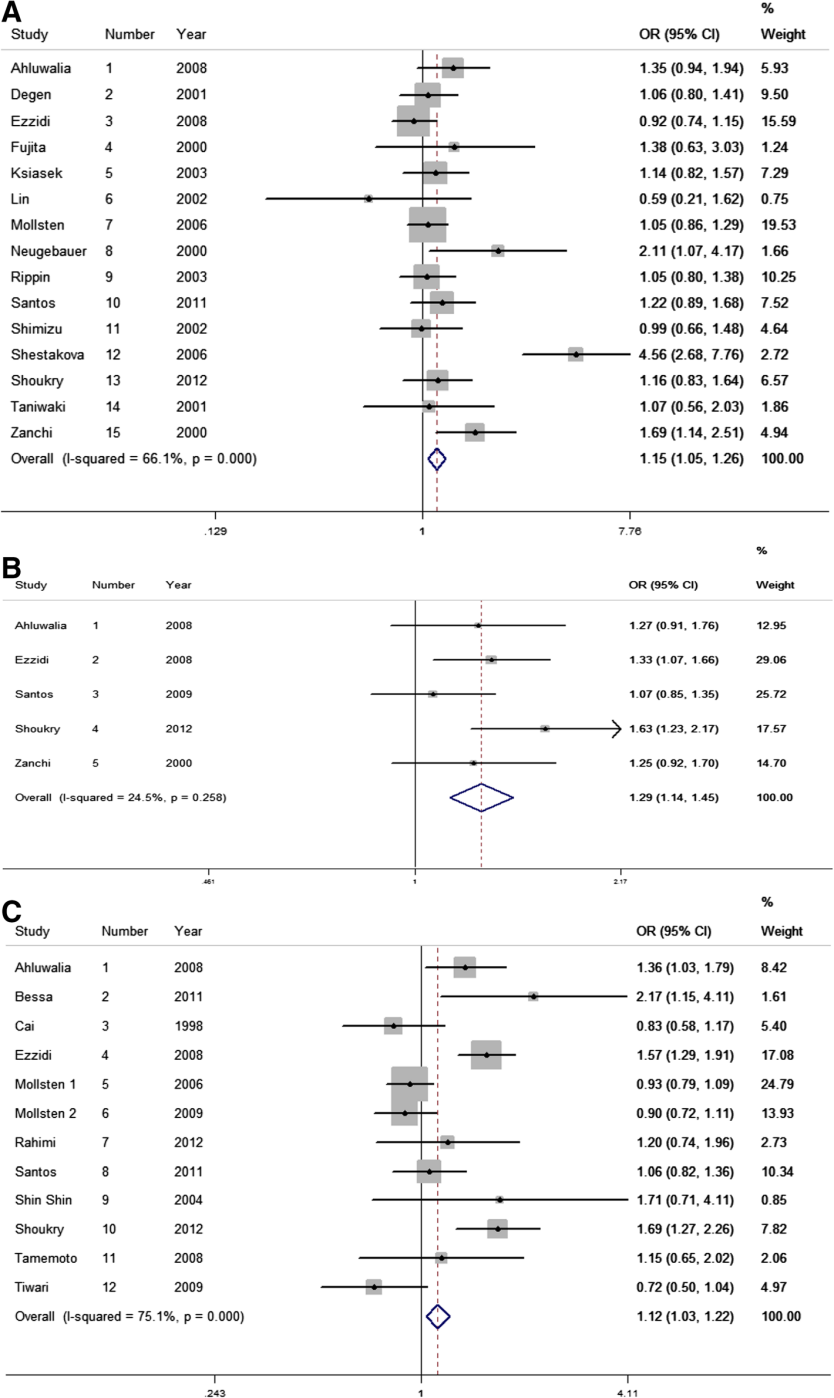
Forest plot for contrast allele model for (A) 4b/a polymorphism; (B) T-786C polymorphism; and (C) G894T polymorphism.

For the 4b/a polymorphism, an association with DN in all genetic models, except for co-dominance, was observed: allele contrast (OR = 1.15, CI (95%): 1.05-1.25, P_Q_ <0.01, I^2^ = 66%); additive (OR = 1.52, CI (95%): 1.18-1.97, P_Q_ <0.01, I^2^ = 62%); recessive (OR = 1.50, CI (95%): 1.16-1.93, P_Q_ <0.01, I^2^ = 64%); and dominant (OR = 1.11, CI (95%): 1.01-1.23, P_Q_ = 0.01, I^2^ = 49%). Similarly, for the T-786C polymorphism the association with DN was found with all models, with exception for co-dominance model: allele contrast (OR = 1.22, CI (95%): 1.07-1.39, P_Q_ = 0.59, I^2^ = 0%), additive (OR = 1.52, CI (95%): 1.18-1.97, P_Q_ < 0.01, I^2^ = 62%), recessive (OR = 1.50, CI (95%): 1.16-1.93, P_Q_ <0.01, I^2^ = 64%) and dominant (OR = 1.11, CI (95%): 1.01-1.23, P_Q_ <0.01, I^2^ = 49%). The G894T polymorphism showed association with DN in allelic contrast (OR = 1.12, CI (95%): 1.03-1.25, P_Q_ <0.01, I^2^ = 75%) and co-dominance model (OR = 1.13, CI (95%): 1.04-1.37, P_Q_ = 0.01, I^2^ = 60%) (Table 3 and Figure 2). A random model analysis was performed confirming the fixed model results.

**Table 3:** **Meta-analysis in all genetic models with all patients and subgroup analysis, in fixed-model analysis, presenting heterogeneity (P**_**Q**_**and I**^**2**^**)**

	**Population**	**Studies**	**OR**	**IC (95%)**	**P**	**P**_**Q**_	**I**^**2**^**(%)**
**4b/a**							
**Allele contrast**	All	15	1.15	1.05-1,25	<0.01	<0.01	66
	African	2	0,98	0.81-1.18	0.88	0,25	22
	East Asians	6	1.21	0.97-1.50.8	0.08	0.29	18
	Whites	7	1.20	1.07-1.34	<0.01	<0.01	80
	Type 1	5	1.17	1.02-1.34	0.02	0.07	54
	Type 2	10	1.12	0.99-1.27	0.07	0.28	18
**Additive**	All	15	1.52	1.18-1.97	<0.01	<0.01	62
	African	2	1,13	0,69-1,81	0.62	0.56	0
	East Asians	6	3.25	1.58-6.68	<0.01	0.31	16
	Whites	7	1.49	1.06-2.08	0.01	<0.01	74
	Type 1	5	2.21	1.50-3.25	<0.01	<0.01	81
	Type 2	11	1.36	0.98-1.88	0.06	0.08	41
**Recessive**	All	15	1.50	1.16-1.93	<0.01	<0.01	64
	Africans	2	1.13	0.69-1.83	0,61	0.83	0
	East Asians	6	3.44	1.68-7.05	<0.01	0.28	21
	Whites	7	1.43	1.03-1.99	0.03	<0.01	75
	Type 1	5	2.19	1.49-3.21	<0.01	<0.01	81
	Type 2	11	1.49	1.07-2.07	0.02	0.08	42
**Dominant**	All	15	1.11	1.01-1.23	0.03	0,01	49
	African	2	0.94	0.75-1.18	0.64	0.24	27
	East Asians	6	1.04	0.81-1.34	0.71	0.44	0
	Whites	7	1.20	1.05-1.36	<0.01	<0.01	67
	Type 1	5	1.22	1.04-1.43	0.01	<0.01	78
	Type 2	11	1.05	0.92-1.20	0.44	0.62	0
**Codominant**	All	15	0.98	0.88-1.09	0.81	0,02	46
	African	2	1.09	0.87-1.38	0.42	0.29	7
	East Asians	6	1.17	0.90-1.55	0.22	0.14	38
	Whites	7	0.90	0.79-1.04	0.16	0.04	54
	Type 1	5	0.94	0.80-1.11	0.46	0.01	68
	Type 2	11	1.01	0.88-1.17	0.80	0.19	26
**T-786C**							
**Allele contrast**	All	5	1.28	1.14-1.44	<0.01	0.25	24
	African	2	1.44	1.21-1.71	<0.01	0.26	19
	Whites	2	1.13	0.94-1.36	0.19	0.44	0
	Type 2	4	1.29	1.13-1.46	<0.01	0.15	42
**Additive**	All	5	1.48	1.14-1.92	<0,01	0.01	67
	African	2	1.43	0.98-2.09	0.05	0.01	84
	Whites	2	1.36	0.93-1.98	0.10	0.18	42
	Type 2	4	1.40	1.06-1.86	0.01	<0.01	73
**Recessive**	All	5	1.38	1,09-1.76	<0,01	0.01	68
	African	2	1.24	0.88-1.76	0.21	0.01	81
	Whites	2	1.39	0.98-1.95	0.06	0.09	0
	Type 2	4	1.27	0.98-1.65	0.06	0.01	72
**Dominant**	All	5	1.21	1,04-1.42	0.01	0.29	18
	African	2	1.39	1.11-1.73	<0.01	0.13	54
	Whites	2	1.05	0.81-1.37	0.70	0.95	0
	Type 2	4	1.24	1.05-1.47	<0.01	0.22	31
**Codominant**	All	5	0.95	0.81-1.11	0.53	0.12	45
	African	2	0.78	0.62-0.98	0.03	0.48	0
	Whites	2	1.15	0.89-1.49	0.28	0.24	25
	Type 2	3	0.90	0.75-1.06	0.20	0.131	15
**G986T**							
**Allele contrast**	All	12	1.12	1.03-1.21	<0.01	<0.01	75
	African	3	1.63	1.39-1.91	<0.01	0.61	0
	East Asian	3	1.33	1.05-1.70	0.01	0.74	0
	Whites	4	0.93	0.84-1.04	0.20	0.67	0
	Type 1	2	0.92	0.80-1.04	0.18	0.18	0
	Type 2	9	1.27	1.15-1.42	<0.01	<0.01	72
**Additive**	All	12	1.19	0.99-1.43	0.05	<0.01	63
	African	3	2.01	1.50-2.94	<0.01	0.27	22
	East Asian	3	1.85	1,05-3.25	0.03	0.59	0
	Whites	4	0.86	0.67-1.10	0.23	0.69	0
	Type 1	2	0.87	0.65-1.16	0.34	0.44	0
	Type 2	9	1.47	1.16-1.86	<0.01	<0.01	63
**Recessive**	All	12	1.16	0.97-1.38	0.09	0.02	52
	Africa	3	1.80	1.31-2.46	<0.01	0.43	0
	East Asian	3	1.73	1.01-2.96	0.04	0.63	0
	Whites	4	0.88	0.69-1.11	0.29	0.62	0
	Type 1	2	0.91	0.69-1.20	0.49	0.31	0
	Type 2	9	1.36	1.08-1.70	<0.01	0.03	53
**Dominant**	All	12	0.99	0.89-1.11	0.92	0.07	45
	African	3	1.46	1.17-1.82	<0.01	0.11	54
	East Asian	3	1.32	0.98-1.79	0.06	0.73	0
	Whites	4	0.93	0.80-1.07	0.31	0.59	0
	Type 1	2	0.89	0.75-1.06	0.19	0.74	0
	Type 2	9	1.19	0.92-1.26	0.35	0.04	57
**Codominant**	All	12	1.03	1.04-1.37	0.01	0.01	60
	African	3	0.92	0.74-1.14	0.45	0.29	18
	East Asian	3	0.89	0.65-1.21	0.48	0.52	0
	Whites	4	1.02	0.89-1.18	0.69	0.44	0
	Type 1	2	1.08	0.91-1.29	0.35	0.32	0
	Type 2	9	0.94	0.82-1.08	0.41	0.34	11

**Figure 3:**
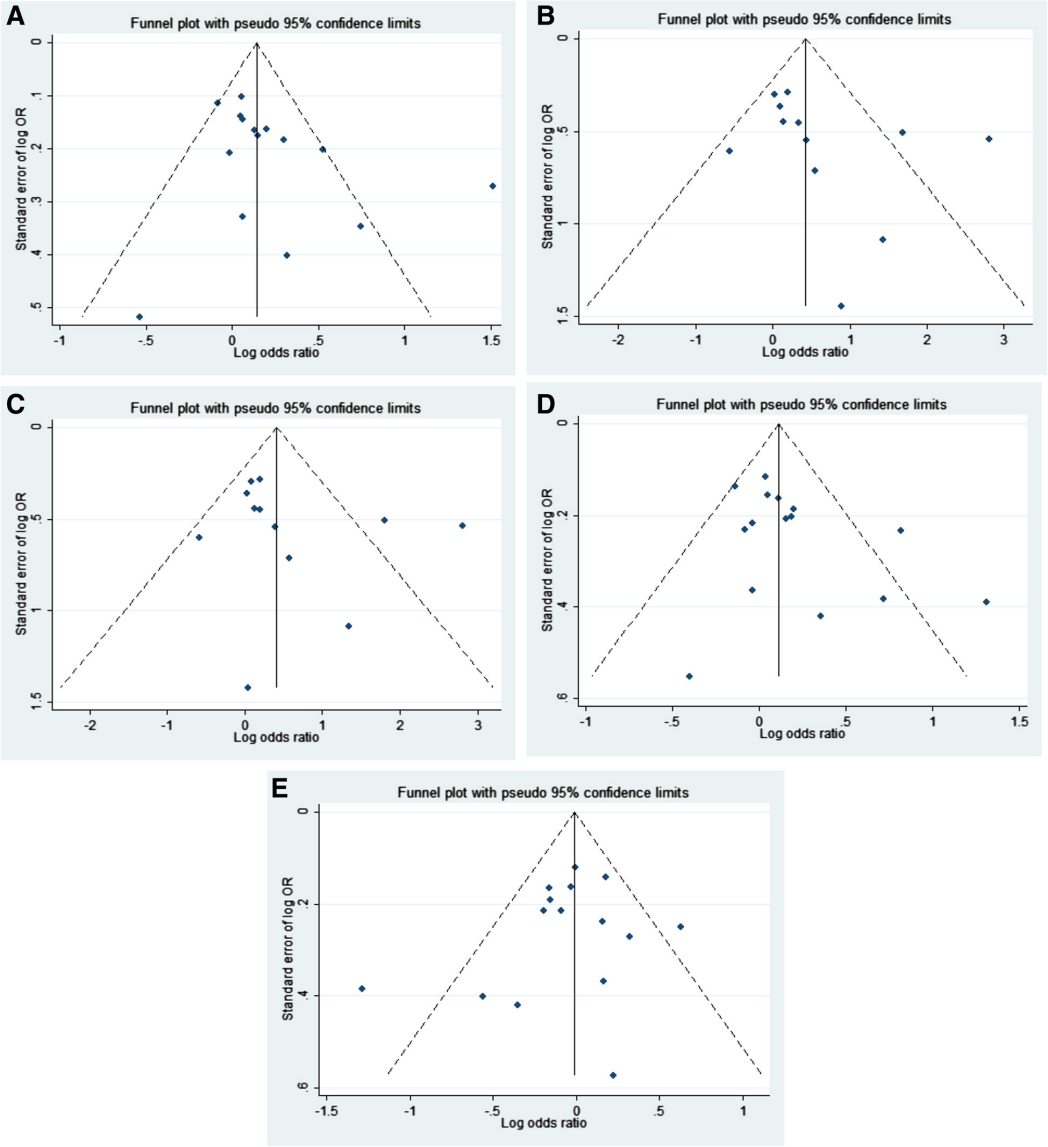
Funnel plot for 4b/a polymorphism: (A) allele contrast; (B) additive; (C) recessive; (D) dominant; (E), codominant.

Publication bias was observed for the majority of the polymorphisms evaluated and are presented as a funnel plot for 4b/a polymorphsism (Figure 3). In order to identify non published data, we performed manual search for abstracts in some of the major scientific meetings in the field in the last seven years. We estimated the effect of these potential publication biases using trim and fill method and no major differences were observed from the original results.

Since some studies included only subjects of specific ethnicities or with type 1 or type 2 DM, we performed a sensitive analysis stratifying the studies according to these characteristics. Considering 4b/a polymorphism, there was an association in White and East Asian populations in allele contrast, additive and recessive models; only for Whites in the dominant model; and none for the co-dominant model. For T-786C variant, no association was shown for Whites in allele contrast analysis or in any other genetic model, but in African populations the polymorphism was associated with DN in allele contrast, dominance and co-dominance models. Considering G894T polymorphism, in African populations the association was observed for all genetic models, with the exception of co-dominance model. There were insufficient studies to perform a meta-analysis for G894T in South Asians and West Asians.

According to the type of diabetes mellitus (DM), for 4b/a polymorphism an association was observed in additive and recessive models for both type 1 and type 2 diabetes, and only for type 1 in allele contrast and dominant models. There was no association with any type of DM in co-dominant model for 4b/a variant. For T-786C, no association in any genetic model was found in type 2 diabetes. There was insufficient data for this analysis in type 1 DM. Likewise, for G894T variant there was an association only in the allele contrast model with type 2 diabetes (Table 3).

We compared the ORs of our meta-analysis with the results from a previous meta-analysis that used non-diabetic patients as controls [30]. The results were similar and no statistical differences in the ORs of the two studies were observed in all genetic models analyzed (data not shown).

## Conclusions

In the present study, the most robust association of DN was with eNOS 4b/a and T-786C polymorphism that held in all genetic models tested, except for co-dominance model. G894T polymorphism was associated with DN only in allele contrast and in co-dominance model. 4b/a polymorphism association with DN was confirmed in all ethnic groups evaluated and for all types of diabetes. The subgroup analysis of the T-786C variant should be viewed with caution, since it was limited due to the small number of studies.

Analyzing genetic model is important, considering the difference between them. Each individual genotype is formed by two alleles (for example G and T for G984T polymorphism), and the risk of every genotype depends on the number of variant allele copies carried, where one of which is thought to be associated with a disease (e.g., T), association studies will collect information on the numbers of diseased and disease-free subjects with each of the three genotypes (GG, GT, and TT). So we used the allele contrast, which compares the number of alleles G with the number of alleles G; the additive model, which contrasts extreme homozygotes, comparing the genotype GG with the genotype TT; in recessive model two copies of T allele are essential to modify the risk, combining the GG and GT genotypes and comparing with TT; the dominant model, which heterozygous GT and homozygous TT genotypes have the similar risk as a single copy of T is sufficient to alter the risk, then compares GG with combined GT and TT genotypes; and the codominance model, commonly used genetic model, where each genotype gives a diverse and non additive risk. which combines the GG and TT genotypes and compares with GT. So OR in each particular genetic model gives us different interpretations about the risk of the polymorphisms.

These results are original and help to understand the role of these polymorphisms in the development of DN. However, it was not possible to exclude a publication bias of negative studies. Therefore, the exact effect could be smaller. As discussed before, other explanations, besides classic risk factors, are needed for understanding the progression of a diabetic patient from normoalbuminuria to macroalbuminuria, and a polymorphism identification of a specific gene would propitiate the development of a new therapy aimed directly to it.

In contrast to a recent meta-analysis performed by Zintzaras et al. [30], which analyzed the same polymorphisms in the progression of DN, our analysis compared diabetic patients with DN (cases) with diabetic patients without DN (controls). In Zintzaras’ study, healthy subjects were used as controls, mixed with patients with DN. When the controls are defined as non-diabetic subjects, the observed association could reflect a genetic predisposition for individuals to develop “diabetic nephropathy”. The obtained results could reflect a mixture of a susceptibility to diabetes per se and to nephropathy, which cannot be discriminated. In this regard, to serve non-diabetic individuals as controls seem rationale to estimate a risk of diabetic nephropathy. However, from clinical points of view, most of medical staff would be interested in risks for nephropathy among individuals with diabetes, as in the case with hyperglycemia, rather than combined risks for developing diabetes and for nephropathy thereafter. That is why diabetic individuals showing no or little nephropathy despite a term of duration have been widely investigated as controls, in most of the previous studies. So, our work and Zintzaras are derived from different standing points: a clinical aspect and a bio-mathematic research.

In this sense, we considered that the optimal control group when studding a DM complication is a diabetic patient without the complication and with disease duration long enough the permit a genetic predisposition to become clinically detected in the presence of hyperglycemia. Moreover, the disease duration must be comparable between cases and controls. Most included studies fulfilled the two pre-requisitions. As can be seen in Table 1, the DM duration is similar between cases and controls in each study and the majority has more than 10 years of DM, reflecting that authors from original studies probably took this important issue in consideration.

Despite the different control used by Zintzaras, they found 92 articles, being 20 included for meta-analysis; that provided 1942/1461 cases/controls for G894T, 2663/2232; cases/controls for 4b/a, and 857/845 cases/controls for T-786C. That was similar to ours that had 22 studies included, but provided about one third more cases/controls. The OR observed in their analyzes showed significance in allelic contrast model for G894 polymorphism, recessive and additive model for 4b/a polymorphism, and allelic contrast model for T-786C, all observed in our study; but our analyze showed association in more genetic models than that, like codominant model for G894T; allele contrast and dominant model for 4b/a; recessive, dominant and additive model for T786C. Furthermore, we compared our ORs with those reported by Zintzaras et al. and no statistical differences were found. With that said, our study reinforce the findings from Zintzaras.

DN development predisposition has not been fully explained, since glycemic control and environmental factors, as well as traditional risk factors, do not accurately predict the occurrence of this diabetic complication in all patients. With this in mind, studies have been trying to resolve this question using genetic approaches. Many candidate genes have been explored in this context, and eNOS polymorphisms have been implicated in the susceptibility to glomerular disease, by mechanisms yet unknown [15]. However, there is no consensus on the role of these polymorphisms in modulation of risk for DN, since the available literature demonstrates mixed results and most of the studies have a small sample. In this scenario, the recommended approach to help investigators in understanding the effect of each polymorphism in DN development is a systematic review and meta-analysis. Our data suggest an association between eNOS polymorphisms and DN. Assuming a recessive model, the relative risk, attributable risk and population attributable risk for the 4a variant ranges are, respectively, 1.20; 0.11; and 0.09.

The present paper has some limitations. The inclusion of studies evaluating patients with DM in several stages of DN, ranging from microalbuminuria to chronic renal insufficiency in kidney replacement therapy, could bias the results due to clinical heterogeneity of cases. Some studies did not present the data separated by DN stages. Furthermore, inclusion criteria in the reviewed studies utilized different methods and cutoffs to define microalbuminuria or macroalbuminuria. Although all clinically validated [35], these aspects made impossible to evaluate the effect of each polymorphism in the stages of DN in this meta-analysis. Finally, the polymorphisms true effects could be overestimated in the present study, since there is some indication of publication bias.

In conclusion, this study shows an association between DN and polymorphisms in eNOS gene. This effect is very consistent for the 4b and T-786 polymorphism.

## Methods

The Preferred Reporting Items for Systematic Reviews and Meta-Analysis (PRISMA) statement was used in this report [36,37].

### Selection criteria and search strategy

Case–control studies that had diabetic patients with DN as cases and diabetic patients without nephropathy as controls, as well as that evaluated at least one of the three polymorphisms of interest (4b/4a, T-786C, G986T) were considered eligible. Only studies in humans and using validated genotyping methods were considered. No publication language, publication date, or publication status restrictions were imposed. All studies published up until December 31^st^, 2012 were identified by searching electronic databases: Medline (1966-Present), EMBASE (1980-Present), LILACS and Cochrane Library.

Abstracts presented at scientific events held by: The American Diabetes Association (ADA); The European Association for the Study of Diabetes (EASD); The National Kidney Association (NKA); and The American Society of Nephrology (ASN) were searched over the last seven years. The authors were contacted for more details in the case of abstracts with missing information.

The following index terms were used: (“Nitric Oxide Synthase Type III” OR “NOS3 protein, human”) AND (“Databases, Genetic” OR “Genetic Predisposition to Disease” OR “Genetic Phenomena” OR “Genetic Processes” OR “Genetic Markers” OR “Genetic Variation” OR “Polymorphism, Genetic” OR “Genetic Research” OR “Genetic Determinism” OR “Genes” OR “Genetics” OR “Mutation” OR “Genetics, Medical” OR “DNA”) AND (“Proteinuria” OR “Albuminuria” OR “Kidney Failure” OR “Kidney Failure, Chronic” OR “Kidney Diseases” OR “Diabetic Nephropathies”).

### Study selection and data extraction

Eligibility assessment was made by title and abstracts review and in doubtful cases by full article review. This was performed independently in a standardized manner by two investigators (BSD and CBL). Disagreements between reviewers were resolved by consensus.

Two investigators extracted the data, one independent to another (BSD and LCFP). Disagreements were resolved by a third author (LHC). For articles with missing information, (n = 3) the authors were contacted for further information, but none responded. In the case of duplicate publications, the first manuscript published was included in the analysis. Information was extracted from each individual study based on: (1) characteristics of study participants (including age, gender, type of diabetes, diabetes duration, nephrologic status, and ethnicity) [38], (2) case and control definition; (3) genetic data (including allelic distribution and genotypic frequency).

### Quality assessment

To ascertain the validity of each eligible case–control study, two investigators (BSD and LCFP) worked independently during the initial search and after worked together to determine the adequacy of studies selection. It was assessed if the same exclusion criteria for cases and controls were used; if cases were easily differentiated from controls; if analysis of studied polymorphisms were conducted in a standard, valid, and reliable way, if major biases were identified and considered in design and analysis; and how good the study was to minimize the risks of bias or confusion. Hardy-Weinberg equilibrium assessment among the control group within each polymorphism in all studies was checked by exact test using an online HWE calculator (http://ihg.gsf.de/cgi-bin/hw/hwa1.pl).

### Statistical analysis

Gene-disease association was measured using odds ratio estimation based on the following genetic contrast/models: (1) allele contrast; (2) additive model; (3) recessive model; (4) dominant model and (4) co-dominant model [39,40]. Heterogeneity was tested by chi-squared test, Cochran’s Q, and inconsistency with I^2^. If P_Q_ <0.10, then heterogeneity was considered statistically significant. Odds ratio was calculated using fixed-effect models (Mantel-Haenszel), and random models when heterogeneity was observed. Multiple comparisons were not made because meta-analysis of genetic association studies is considered an exploratory study, without a prespecified key hypothesis [41,42].

The risk of publication bias was evaluated using funnel plot graphics [43].

Sensibility tests were made concerning to ethnia and type of diabetes.

Data were analyzed using Stata/SE 11.2 (http://www.stata.com).

We compared the ORs of our meta-analysis with the results from a previous one [44] that used non-diabetic patients as controls using the differences of OR and 95%CI (WinPepi version 11.3).

## Abbreviations

ADA: The American Diabetes Association; ASN: The American Society of Nephrology; DM: Diabetes mellitus; DN: Diabetic nephropathy; EASD: The European Association for the Study of Diabetes; eNOS: Endothelial nitric oxide synthase; ESRD: End stage renal disease; GFR: Glomerular filtration rate; iNOS: Inducible nitric oxide synthase; NKA: The National Kidney Association; nNOS: Neuronal nitric oxide synthase; NO: Nitric oxide; NOS: Nitric oxide synthase; PRISMA: The preferred reporting items for systematic reviews and meta-analysis; SNP: Single nucleotide polymorphism.

## Competing interests

The authors declare that they have no competing interest.

## Authors’ contributions

BSD participated in design, selection of included articles, data collection, statistical analysis and wrote the manuscript; LCFP participated in data collection; CBL participated in selection of included articles and wrote the manuscript; KGS data collection and wrote the manuscript, LHSC wrote the manuscript. All authors read and approved the final manuscript.

## Pre-publication history

The pre-publication history for this paper can be accessed here:

http://www.biomedcentral.com/1471-2350/15/9/prepub
